# Does Mandala Art Improve Psychological Well-Being in Patients? A Systematic Review

**DOI:** 10.1089/jicm.2022.0780

**Published:** 2024-01-12

**Authors:** Meng-Qin Zhang, Xing Liu, Yan Huang

**Affiliations:** ^1^ Department of Gynecological Nursing, West China Second University Hospital, Sichuan University/West China School of Nursing, Chengdu, China.; ^2^ Key Laboratory of Birth Defects and Related Diseases of Women and Children (Sichuan University), Ministry of Education, Chengdu, China.; ^3^ Department of Nursing, West China Second University Hospital, Sichuan University/West China School of Nursing, Chengdu, China.

**Keywords:** art therapy, mandala art, patient, psychological well-being, systematic review

## Abstract

**Objectives:**

Psychological disorders and symptoms are common and can significantly impair functioning in some areas in patients. We aimed to investigate the effects of mandala art (MA) on psychological well-being in patients.

**Design:**

A systematic review.

**Methods:**

We did a systematic review to assess the associations between MA and psychological well-being among patients. A systematic search of PubMed, EMBASE, Web of Science, CINAHL, PsycINFO, and Cochrane Central Register of Controlled Trial databases was conducted by independent reviewers from database inception to April 2023. We include randomized controlled trials, quasi-experimental studies, and qualitative studies. Outcome measures included any measurement of subjective and objective psychological well-being outcomes, such as stress, anxiety, depression, distress, mindfulness, hope, resilience, pain, mood, fatigue, and trauma symptoms.

**Results:**

Eleven studies of 405 participants were identified in the systematic review. Overall, the included studies provided preliminary evidence to suggest that MA may improve negative symptoms and hope, relieving pain, and reducing some physiological indicators of stress in patients. However, the quality of the existing evidence limited the generalization of results.

**Conclusions:**

According to the current evidence, the therapeutic benefits of using mandalas for improving the psychological well-being of patients are uncertain. More well-designed and high-quality studies in the field of MA are needed in the future.

## Introduction

The prevalence of psychological problems is generally high, and psychological disorders and symptoms have a significant effect on some aspects of functioning. It is reported that up to 30% of the general population suffered from mental disorders.^1^ In 919 patients recruited from two primary care clinics, almost 40% reported anxiety symptoms and 30% reported depression symptoms.^2^ Most patients, whether they are hospitalized or at home, experience anxiety and/or depression symptoms due to physical illness, which can lead to cardiovascular disease,^3,4^ mortality,^5,6^ and poorer quality of life.^2^ Approximately 30% of patients with cancer experience distress in disease progression.^7,8^ The presence of distress negatively impacts patients' social skills, quality of life, survival rates, and ability to deal with cancer.^9^ As a result of distress, cancer incidence increases by 13% and cancer mortality increases by 27%.^10^ It has been reported that more than half (59%) of cancer patients experience high levels of anxiety.^11^ In a review of 110 studies, 36% of children and adolescents with cancer reported psychological problems and symptoms.^12^ Moreover, the coronavirus disease 2019 (COVID-19), as an emerging disease, has widespread effects on both the physical and mental health of individuals.^13^ Commonly experienced negative emotions include anxiety, depression, helplessness, confusion, fear, and guilt.^14,15^

There are various pharmacological and nonpharmacological approaches available to address physical and psychological distress in patients. However, pharmacological approaches are usually associated with side effects causing clinical deterioration. Administering benzodiazepines or other pharmacological agents for anxiety treatment increased the prevalence of respiratory depression and other side effects; the use of tricyclic antidepressants to treat depression was associated with a higher incidence of orthostatic hypotension,^16^ ventricular arrhythmia, and sudden cardiac death.^17^ As a result, nonpharmaceutical interventions, also known as complementary and alternative therapies, may be safer options. The Mandala art (MA) intervention is an art therapy approach that is being used in complementary and alternative medicine to manage physical and psychological symptoms, improve psychological distress, relieve the level of anxiety, and promote relationships.^18–22^

Art therapy, which involves art as a supplementary therapy for neurological, mental, or behavioral disorders, has grown in popularity since the 1960s. Art therapies usually include dance movement therapy, music therapy, poetry therapy, drama therapy, and expressive art therapies, which generally combine different art forms. Art therapies have achieved significant therapeutic effects on psychological well-being in both clinical and nonclinical settings over the past few decades. The mandala is considered a psychotherapy technique that provides psychological support and healing by art psychotherapists.^23^

In Sanskrit, a mandala represents a circle or center. It is often associated with Eastern religions and philosophies, such as Buddhism and Tibetan Buddhism.^24^ Carl Jung, an important psychotherapist, used the mandala technique to heal or solve problems in the early 1900s; the resulting mandala drawing reflects one's self-perception and current mood.^25^ The use of mandala is classified into two types: drawing free figures in a circle (unstructured mandala), or coloring a given pattern in a circle (structured mandala).^26^ Mandala, as a form of art therapy, has been gradually recognized as a useful intervention that can be used by various individuals and in many settings.

MA interventions have been proven to be effective in reducing anxiety in breast cancer patients^18^; increasing hope of psychiatric inpatients^27^; lowering stress in emergency medicine providers and medical–surgical nurses^21,28^; easing the burdens of care and stress for family caregivers and oncology professionals^29^; and reducing anxiety and processing complex personal emotions in college students.^30–32^ In addition, previous studies have used MA to reduce pain and anxiety, identify psychological problems, promote concentration, and provide psychological comfort to children and adolescents.^19,33–35^ However, there is some evidence suggesting that not all individuals respond favorably to MA. Previous studies reported that MA had no effects on depression,^36^ stress,^21^ fatigue,^20^ resilience, and subjective well-being ^27^ in patients; even increased distress scores in cancer survival patients.^18^

The effectiveness of art therapy and painting therapy in reducing psychological negative symptoms has been reviewed. However, to our knowledge, there have been no published systematic reviews of the effectiveness of MA intervention on psychological well-being and no overview of the intervention characteristics until now. In addition, there are inconsistencies in the research results of MA interventions. Considering the safety and accessibility of MA, no need for special equipment, and also less interference from therapists through the treatment process, this systematic review was conducted to investigate the effect of MA on mental well-being among patients.

## Method

The systematic review was carried out according to the Cochrane Handbook (version 5.1.0). The PRISMA guidelines were followed for reporting. See Supplementary file S1 for the details of PRISMA. This review was not registered at PROSPERO. This systematic review is a secondary research based on some previously published data. The ethical approval and informed consent were not required for this study as this review did not involve humans or animals.

### Selection criteria

Publications in English were included with no restriction on publication date. The inclusion criteria were:

Study type: randomized controlled trials (RCTs), quasi-experimental studies, case reports, and qualitative studies.

Patients: participants who reported some psychological problems, were diagnosed with physical diseases according to standardized diagnostic criteria, and were undergoing active treatment regardless of age, gender, ethnicity, or disease type.

Intervention: any type of MA intervention (such as structured mandala or unstructured mandala) provided to individuals or groups, regardless of the intervention frequency and duration.

Comparison: involved one of the following treatments: receiving no treatment, standard clinical care/treatment-as-usual (TAU), or any other active treatment.

Outcomes: measurement of subjective and objective psychological well-being outcomes, such as stress, anxiety, depression, distress, mindfulness, hope, resilience, pain, mood, fatigue, and trauma symptoms.

Exclusion criteria: studies that assessed the reduction of psychological symptoms in healthy populations and those that artificially induced psychological symptoms in individuals.

### Search strategy and information sources

From database inception to April 26, 2023, we searched PubMed, Embase, Web of Science, CINAHL (using the Ovid platform), PsycINFO (using the Ovid platform), the Cochrane Library, ClinicalTrials.gov, and World Health Organization (WHO) International Clinical Trials Registry Platform. References of included studies and relevant systematic reviews were also screened. MeSH terms, Emtree terms, and Psychological Index terms corresponding to each database were used when applicable. The search terms used to search each database were: Art therapy, mandala, mandala art, mandala art therapy, mandala coloring, mandala-coloring, mandala drawing, mandala painting, mindfulness-based coloring, mindful coloring, deliberative coloring, coloring, structured coloring, therapeutic coloring, therapeutic artmaking, art-based mandala, painting, drawing) AND (Stress or Anxiety or Depression or Distress or Mindfulness or Hope or Resilience or Pain or Fatigue or mood or Mental disorders or wellbeing or trauma symptoms) AND (Patients or cancer or disability). The detailed search strategy in PubMed can be found in Supplementary file S2.

### Data screening and extraction

As we focused on peer-reviewed raw data, duplicate publications, thesis, and conference abstracts were removed. The screening of the articles was undertaken by two independent reviewers (M.-Q.Z. and X.L.). The titles and abstracts of the entries were screened for eligibility and the full texts of the screening results were downloaded for the second screening. Any disagreement in the screening process was resolved by consensus or by the third reviewer (Y.H.).

The data were extracted by two researchers independently. The items of the data extracted mainly included: first author, publication year, study design, region, sample size, population, intervention and comparison measures, duration time, intervention session, intervention implementer, outcome measure, and main outcomes.

### Assessment of quality

The quality of the studies was assessed independently by two reviewers (M.-Q.Z. and X.L.), and any divergence of opinions was resolved by consensus or involvement of a third reviewer (Y.H.). The Joanna Briggs Institute (JBI) Critical Appraisal tools^37^ for RCT, quasi-experimental study, and qualitative study were used to assess the risk of bias in each study.

### Statistical analysis

There was considerable heterogeneity in the included studies. As a result of heterogeneity in outcome reported and study design, a meta-analysis could not be conducted. Accordingly, a narrative synthesis of the results of RCTs, quasi-experimental studies, and qualitative studies was executed.

## Results

### Study characteristics

**FIG. 1.**
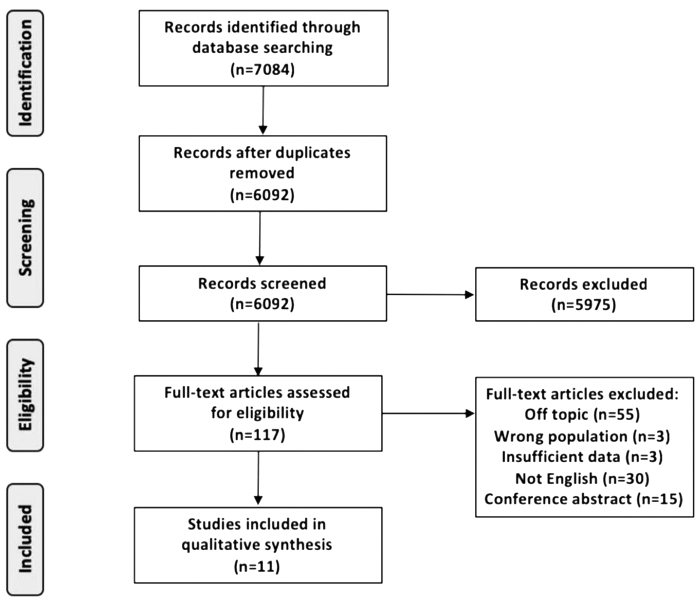
Study selection flow diagram.

The study selection process is shown in Figure 1. In total, 7084 studies were identified through the electronic search. After removing duplicates, 6092 studies remained for titles and abstracts screening. A total of 117 articles were obtained after reading the titles and abstracts. Of these, 11 studies met the review criteria and were finally included after reading the full text. The included studies were published from 2011 to 2023, including six RCTs, four quasi-experiment studies, and one qualitative research study. The general patient characteristics of each article are presented in Table 1.

**Table 1.** Characteristics of Studies

Author, year	Study design	Region	*n *(EG/CG)	Patient	Intervention	Intervention duration	Intervention session	Intervention implementer	Control	Outcome measure	Results
Gürcan and Atay Turan (2021)^38^	RCT	Turkey	60 (30/30)	Hospitalized adolescents with cancer	Mandala drawing	5–6 days	Two sessions, for ∼1–2 h each	Not mentioned	TAU	1. The Hospital Anxiety and Depression Scale 2. The Memorial Symptom Assessment Scale (Psychological subscale)	The anxiety and depression scores, and the psychological symptom scores significantly decreased in the intervention group, compared with the CG
Khademi et al. (2021)^22^	RCT	Iran	70 (35/35)	Hospitalized patients with COVID-19	Mandala coloring	6 consecutive days	Six sessions, for 30 min each	Not mentioned	TAU	The Spielberger STAI	The anxiety scores significantly decreased in the intervention group as compared with the CG
Choi et al. (2021)^20^	RCT	Korea	36 (21/15)	Building and facilities management employees with chronic widespread musculoskeletal pain	MBMC coloring	4 h	Single session	Registered art psychotherapist	A nonguided sightseeing bus tour	1. The Manual Tender Point Survey 2. The Fatigue Severity Scale 3. The Stress Response Inventory-Modified Form 4. Stress hormone cortisol	Significant improvements in tender points, total stress level, depressive symptoms, anger symptoms, and salivary cortisol in the MBMC group
Czamanski-Cohen et al. (2019)^39^	RCT	Israel	15 (8/7)	Breast cancer patients	Mandala coloring	8 weeks	Eight sessions, 1 day a week, for 1 h each	Art therapist	Art therapy	1. The Levels of Emotional Awareness Scale 2. The AE Scale 3. The Center for Epidemiologic Studies-Depression scale 4. Physical symptoms: the Breast Cancer Prevention Trial Symptom Scale	Statistically significant between-group differences in emotional awareness and AE were found. Large effect sizes between groups and over time in AE, emotional awareness and depressive symptoms
Stinley et al. (2015)^19^	RCT	United States	40 (20/20)	Pediatric patients undergoing venipuncture procedure	Creating a mandala by coloring or drawing anything inside or outside the circle on an iPad	30 min	Single session	Not mentioned	TAU	1. Heart rate and blood oxygen saturation 2. The Hospital Fears Rating Scale 3. The Wong–Baker visual analog scale pain instrument 4. Behavioral Indicators of Stress (e.g., fidgeting, crying, screaming, physical struggle)	Physiological stress behaviors were significantly reduced in the Treatment Group compared with the CG. Psychological anxiety decreased significantly in Treatment Group participants
Schrade et al. (2011)^40^	RCT	United States	18 (6/6/6)	Individuals with intellectual disability	Mandala making	15 min	Single session	Occupational therapist	Free drawing, and a neutral control condition: provided puzzles and/or table games	1. Systolic blood pressure, diastolic blood pressure, and/or pulse	Blood pressure change in the mandala making condition indicated a statistically significant reduction in both diastolic and systolic pressure between the first and third reading
Akbulak and Can et al. (2023)^41^	Quasi-experimental study	Turkey	84 (41/43)	Women with early-stage breast cancer receiving chemotherapy for the first time	Mandala coloring	30 min	Single session	Not mentioned	TAU	1. The Distress Thermometer 2. The STAI	Patients in the intervention group who had high distress levels before premedication showed a significant decrease in state anxiety score after premedication
Yakar et al. (2021)^18^	Quasi-experimental study	Turkey	12	Breast cancer patients	Art-based mandala intervention combined with different meditation techniques	8 weeks	Eight sessions, 1 day a week, for 2 h each	Mandala meditation therapy specialist	/	1. The Distress Thermometer 2. The 20-item Spielberger STAI-State Anxiety Scale (STAI-S)	Anxiety scores decreased significantly after the program compared with before the program. The distress scores increased after the program compared with before the program
Barati et al. (2020)^36^	Quasi-experimental study	Iran	30 (15/15)	Multiple sclerosis patients	Software-based therapeutic coloring (mandala coloring)	4 weeks	Twelve sessions, 3 sessions per week	Not mentioned	TAU	1.The Depression Questionnaire 2.The Depression Anxiety Stress Scale (DASS-21)	Anxiety score was significantly lower in the intervention group as compared with the controls. A significant reduction in score of stress was observed in the intervention group as compared with preintervention. Stress score significantly reduced more in the intervention group as compared with the control
Kim et al. (2017)	Quasi-experimental study	Korea	28 (15/13)	Psychiatric inpatients	Mandala art therapy (mandala coloring)	4 weeks	Eight sessions, two sessions per week, for an hour each	Mandala art therapist	TAU	1. The Concise Measure of Subjective Well-being 2. The Resilience Scale 3. The Schizophrenia Hope Scale-9	Hope significantly increased in both groups, but the overall increase was greater in the EG at pre- and post-test, respectively than in the CG
Gürcan and Atay Turan. (2020)^35^	Qualitative study	Turkey	12	Adolescents with cancer	Unstructured mandala drawing	1–2 h	Single session	Not mentioned	/	Analyze the mandala drawing and the interview texts	Two main themes with related subthemes each were obtained: being an adolescent with cancer, with the subthemes of changes in health, restriction of freedom and feeling lonely; and coping with cancer with the subthemes of psychological growth and hope for healing

AE, Acceptance of Emotions; CG, control group; COVID-19, coronavirus disease 2019; EG, experimental group; MBMC, mindfulness-based mandala; RCT, randomized controlled trial; STAI, State-Trait Anxiety Inventory; TAU, treatment-as-usual.

### Study designs and participants

Among the eligible studies in this review, intervention durations ranged from 15 min to 8 weeks, with 1 to 12 sessions in total. In addition, each session duration differed from 15 min to 4 h. In two studies, the intervention group receives the cointervention: meditation techniques^18^ and mindfulness-based intervention,^20^ respectively. Only two studies included software-based intervention.^19,36^ The intervention groups included structured mandala and unstructured mandala. The control groups included TAU, receiving no treatment, and active control. In terms of measurement, the outcome measures used in the included studies differed. Tools used to measure anxiety include the State-Trait Anxiety Inventory,^18,22^ the Hospital Anxiety and Depression Scale (HADS),^38^ and the Hospital Fears Rating Scale.^19^ The outcome measures for depression included the Center for Epidemiologic Studies-Depression Scale,^39^ and the HADS.^38^ The outcome measures for stress included the Stress Response Inventory-Modified Form^20^; the specific physiological stress response reflected in changes in vital signs^19,40^ and stress hormone cortisol.^20^

The outcome measures for pain differed and included the Wong–Baker visual analog scale pain instrument^19^ and the Manual Tender Point Survey.^20^ Moreover, the Depression Anxiety Stress Scale,^36^ a comprehensive scale of multiple subscales, was used to assess the negative effect of depression, anxiety, and stress. Of the other measurements, the Fatigue Severity Scale, the Distress Thermometer, the Levels of Emotional Awareness Scale, the Acceptance of Emotions (AE) Scale, the Concise Measure of Subjective Well-being, the Resilience Scale and the Schizophrenia Hope Scale-9 were used to assess related well-being outcomes.

Among the 11 studies, 3 included patients with breast cancer, 2 recruited adolescents receiving cancer therapy, 1 recruited pediatric patients undergoing venipuncture procedure, and 5 studies with other diseases (chronic widespread musculoskeletal pain, intellectual disability, multiple sclerosis, COVID-19, and psychiatric diseases). The sample sizes of included studies ranged from 12 to 84. The total number of participants from all included studies was 405.

### Intervention characteristics

The total duration of the mandala interventions varied from 15 min to 8 weeks, while the single intervention length ranged from 15 min to 4 h per session. Additionally, the number of total sessions ranged from 1 to 12 sessions, and 3 of the interventions were 8 sessions in length, 1 was 12 sessions, 1 was 6 sessions, 1 was 2 sessions, and 5 were single session. The MA interventions included coloring a predesigned mandala and an unstructured mandala drawing. Seven studies used mandala coloring, and three used unstructured mandala drawing; the remaining one did not describe it clearly. Two studies were conducted based on electronic media, using iPad and mobile phones, respectively. In two studies, MA was combined with another treatment: meditation techniques and mindfulness-based intervention, respectively. The remaining studies solely concerned MA. Moreover, two studies reported that MA interventions were conducted by the MA therapist, two were conducted by the art therapist, one was conducted by the occupational therapist, and the remaining six did not mention whether the therapist participated in the research.

### Outcomes of individual studies

There were six RCTs that studied the effects of MA in patients, one RCT^20^ showed significant improvements in tender points, total stress level, depressive symptoms, anger symptoms, and salivary cortisol for patients with chronic widespread musculoskeletal pain. Czamanski-Cohen et al.^39^ found significant differences between groups in emotional awareness and AE for women with breast cancer. The research of Gürcan and Atay Turan^38^ demonstrated that the use of mandala drawing improved anxiety and depression in hospitalized adolescents with cancer. Khademi et al.^22^ identified that coloring the mandala had a positive effect on reducing anxiety in hospitalized patients with COVID-19. Stinley et al.^19^ found a significant reduction in physiological stress behaviors and within-group differences in psychological anxiety in pediatric patients undergoing venipuncture procedures. According to Schrade et al.,^40^ results were inconclusive because no between-group outcomes were provided, but did find a significant reduction of blood pressure in the MA group as compared with preintervention for intellectual disability patients.

Of four quasi-experiment studies, Yakar et al.^18^ found that art-based mandala intervention might be effective in reducing anxiety symptoms, but increasing distress symptoms of breast cancer women. Barati et al.^36^ identified that software-based mandala coloring had positive effects on reducing anxiety and stress states for multiple sclerosis patients. The research of Kim et al.^27^ demonstrated that the use of MA might have effectively increased hope in psychiatric inpatients; by contrast, there were no significant differences in subjective well-being and resilience. Findings of research conducted by Akbulak and Can^41^ suggested that mandala coloring may alleviate anxiety in patients who had high distress levels.

The qualitative research conducted by Gürcan and Atay Turan^35^ indicated that unstructured mandala drawing and interview strategies might be a tool in facilitating communication and understanding psychological growth and hope of cancer adolescents receiving chemotherapy.

### Risk of bias and quality assessment

Based on the JBI Critical Appraisal Tools^42^ for assessing the risk of bias and methodological quality, estimations of bias were made. Since the 11 eligible studies included varied in study design, different quality appraisal methods were required according to the JBI Critical Appraisal Tools. Table 2 summarizes the risk of bias associated with the included six RCTs. Table 3 shows the estimations of bias of the included four quasi-experiments. The assessment results of one qualitative research study are listed in Table 4.

**Table 2.** Risk of Bias and Quality Assessment of Randomized Controlled Trial

Item	Gürcan and Atay Turan^38^	Khademi et al.^22^	Yes/no/unclear/not applicable			
			Choi et al.^20^	Czamanski-Cohen et al.^39^	Stinley et al.^19^	Schrade et al.^40^
1. Was true randomization used for assignment of participants to treatment groups?	Unclear	Yes	Unclear	Unclear	Yes	Unclear
2. Was allocation to treatment groups concealed?	Yes	Unclear	Unclear	Unclear	Unclear	Unclear
3. Were treatment groups similar at the baseline?	Yes	Yes	Yes	Unclear	Unclear	Yes
4. Were participants blind to treatment assignment?	No	No	No	No	No	No
5. Were those delivering treatment blind to treatment assignment?	No	No	No	No	No	No
6. Were outcomes assessors blind to treatment assignment?	No	Unclear	Yes	Unclear	Unclear	Unclear
7. Were treatment groups treated identically other than the intervention of interest?	Yes	Yes	Yes	Unclear	Yes	Yes
8. Was follow-up complete and if not, were differences between groups in terms of their follow-up adequately described and analyzed?	Yes	Yes	Yes	No	Yes	Yes
9. Were participants analyzed in the groups to which they were randomized?	Yes	Yes	No	No	Yes	No
10. Were outcomes measured in the same way for treatment groups?	Yes	Yes	Yes	Yes	Yes	Yes
11. Were outcomes measured in a reliable way?	Yes	Yes	Yes	Yes	Yes	Yes
12. Was appropriate statistical analysis used?	Yes	Yes	Yes	Yes	Yes	Yes
13. Was the trial design appropriate, and any deviations from the standard RCT design (individual randomization, parallel groups) accounted for in the conduct and analysis of the trial?	Yes	Yes	Yes	Yes	Yes	Yes

RCT, randomized controlled trial.

**Table 3.** Risk of Bias and Quality Assessment of Quasi-Experiment Study

Item	Akbulak and Can^41^	Yes/no/unclear/not applicable		
		Yakar et al.^18^	Barati et al.^36^	Kim et al.*^59^*
1. Is it clear in the study what is the “cause” and what is the “effect” (i.e., there is no confusion about which variable comes first)?	Yes	Yes	Yes	Yes
2. Were the participants included in any comparisons similar?	No	Not applicable	No	Unclear
3. Were the participants included in any comparisons receiving similar treatment/care, other than the exposure or intervention of interest?	Yes	Not applicable	Unclear	Unclear
4. Was there a control group?	Yes	No	Yes	Yes
5. Were there multiple measurements of the outcome both pre and post the intervention/exposure?	Yes	No	Yes	Yes
6. Was follow-up complete and if not, were differences between groups in terms of their follow-up adequately described and analyzed?	Yes	No	Yes	No
7. Were the outcomes of participants included in any comparisons measured in the same way?	Yes	Yes	Yes	Yes
8. Were outcomes measured in a reliable way?	Yes	Yes	Yes	Yes
9. Was appropriate statistical analysis used?	Yes	Yes	Yes	Yes

**Table 4.** Risk of Bias and Quality Assessment of Qualitative Study

Item	Yes/no/unclear/not applicable
	Gürcan and Atay Turan^35^
1. Is there congruity between the stated philosophical perspective and the research methodology?	Yes
2. Is there congruity between the research methodology and the research question or objectives?	Yes
3. Is there congruity between the research methodology and the methods used to collect data?	Yes
4. Is there congruity between the research methodology and the representation and analysis of data?	Yes
5. Is there congruity between the research methodology and the interpretation of results?	Yes
6. Is there a statement locating the researcher culturally or theoretically?	Yes
7. Is the influence of the researcher on the research, and vice-versa, addressed?	Unclear
8. Are participants, and their voices, adequately represented?	Yes
9. Is the research ethical according to current criteria or, for recent studies, and is there evidence of ethics approval by an appropriate body?	Yes
10. Do the conclusions drawn in the research report flow from the analysis, or interpretation, of the data?	Yes

## Discussion

Due to heterogeneity in the study population, specific intervention methods, control treatments, and the use of different measurements, the data between the included studies were not sufficiently comparable, so a meta-analysis was not performed. Consequently, the studies were qualitatively described. We performed a systematic review to evaluate the effects of MA on psychological distress in patients. Studies have identified positive results of MA based on current evidence. Specifically, (1) MA might benefit patients concerning negative symptoms, such as anxiety,^18,19,22,36,38,41^ depression,^20,38^ stress,^20,36^ and fear.^19^ (2) MA could relieve chronic pain in patients with chronic widespread musculoskeletal pain,^20^ and acute pain in pediatric patients undergoing venipuncture procedure.^19^ (3) MA had a positive effect on hope in psychiatric inpatients.^27^ (4) MA was effective in reducing some physiological indicators of stress, such as stress hormone cortisol,^20^ blood pressure,^40^ and heart rate.^19^

In this review, MA has been shown to promote physical health and emotional well-being in patients. When searching for relevant studies on this topic, it can be seen that MA interventions are typically designed to relieve the anxiety and stress experienced by students. According to Ramos Salazar,^43^ it reduced anxiety about mathematics courses in business school students and based on findings from Sandmire et al. and Gebhart et al., it reduced first-year college students' anxiety before the final exams^44^ and university nursing students' test anxiety and stress.^45^ In addition, research demonstrated that MA reduces artificially induced anxiety or negative mood symptoms in students.^46–48^

There is a noticeable heterogeneity in the interventions: seven studies used mandala coloring,^18,20,22,27,36,39,41^ three used unstructured mandala drawing,^35,38,40^ and the remaining one did not describe clearly.^19^ MA was also combined with different meditation techniques^18^ and mindfulness-based intervention^20^ in two studies, respectively. There was also a clear difference in the duration and frequency of interventions among the studies included in this review. Researchers do not seem to have sufficiently examined what dose, in other words, what frequency, duration, and length, is necessary to achieve positive effects. It is unclear whether or not the results of different MA therapy interventions can be compared. Since the intervention dose is one of the most important factors in behavioral interventions, this is of particular importance in future work.^49^

Only a little research is available into the psychological mechanisms through which psychotherapeutic interventions create change due to the complexity of study design and implementation.^39^ MA can improve psychological well-being by enhancing self-expression, self-concentration, and self-healing ability. Mandala is an art therapy method that allows individuals to express themselves within a trusting relationship^50^; mandala is also a clinical tool to convert the attention of patients from the medical procedures and transfer one's feelings, perceptions, and beliefs through coloring or drawing.^51^ Ramos Salazar^43^ stated that the use of symbols in mandala allows the expression of internal feelings and psychological traumas, which are often hard to express in front of others. Patients are able to concentrate on the action of coloring or drawing instead of the chaos they are experiencing at the moment.^24,52^ When decorating circles in the mandala, individuals forgot their past negative experiences, expressed their current feelings, and placed themselves at the center of their lives.^23,53^

According to the America Art Therapy Association, art therapists must obtain the minimum of a master's degree and complete the minimum clock hours of supervised clinical practicum and internship programs; psychology and creative arts are included in advanced graduate studies.^54^ Furthermore, an art therapist integrates the fields of art, creative expression, education, counseling, community work, and marriage and family therapy into a unique profession,^55^ which requires a high level of knowledge reserve and competence. How art therapy is conveyed depends on how it is perceived.^55^ Therapist factors seem likely to be confounding the exact effects of research results. However, five of the included studies reported that MA interventions were conducted by the art therapist, and the remaining six did not report whether the therapist was involved in the research.

Two studies were conducted based on electronic media, using iPad and mobile phones. In Stinley's study,^19^ children were asked to create a mandala by coloring or drawing anything inside or outside the circle on an iPad circle template, aiming to avoid excluding patients due to difficulty in fine motor movements. Barati et al.^36^ installed mandala designs coloring software on the included patients' mobile phones, providing three mandala designs for participants to choose from. Both studies have demonstrated the benefits of mandalas on the patient's psychological distress. In the age of information and technologies, mobile terminals such as iPad and mobile phones have shown great development potential in the field of chronic disease management and telemedicine due to their convenience, simplicity, and interactivity. It is suggested that electronic media-based MA interventions can be carried out in clinical practice.

With the aid of portable electronic equipment, such as iPad and mobile phones, MA interventions can not only be implemented in hospitals, but also be extended to homes, communities, and long-term care facilities, thus getting rid of the shackles of face-to-face communication, and greatly improving the accessibility, convenience, and cost-effectiveness of the mandala.

Additionally, the computer assessment systems and estimation systems of MA are gradually developing for the analysis of psychological disorders. In creative art therapies, Gantt^56^ emphasized the need to develop reliable and valid standardized instruments. Suitable computer assessment systems have been developed by Kim. According to Kim et al.,^57^ a computer assessment system can be applied to rate the elements, accuracy, completeness, and concentration of the given pattern coloring in the structured mandala, but the severity of the psychological disorder was not estimated. Furthermore, Kim extended the results to the analysis of psychological disorders. Based on the elements in the structured mandala, Kim et al.^26^ developed another computer system to estimate patients' levels and severity of dementia, which was found to provide art therapists with valuable insight into dementia stages. Computer systems enrich and promote the application of MA. Whether nonexperts like nurses, teachers, guardians, or parents can apply such systems to detect psychological problems early and seek professional treatment is unclear.

## Limitations

Our systematic review has several limitations. First, the sample size of included studies was small. Second, the lack of high-quality RCTs and quasi-experiment studies limited the generalization of results. Third, some eligible studies reflected bias, such as publication bias, and the lack of blinding of patients and personnel; however, Grant et al.^58^ recommend that tools that assess the study quality and bias should be tailored to art therapy where blinding is inaccessible. Fourth, although all eligible studies involved MA, MA interventions are highly heterogeneous, limiting the possibility of generalizing results.

Considering the relatively low quality of current evidence, the therapeutic benefits of using mandalas for improving the psychological well-being of patients are uncertain. More well-designed and high-quality studies in the field of MA are needed in the future.

## Data Availability Statement

Data sharing is not applicable to this study as no new data were created or analyzed.

## Supplementary Material

Supplemental data

Supplemental data
