# Supplementary material for: Does Mandala Art Improve Psychological Well-Being in Patients? A Systematic Review
Source: J Integr Complement Med. 2024 Jan 12;30(1):25–36. doi: 10.1089/jicm.2022.0780 (PMC10801676; doi:10.1089/jicm.2022.0780)
Supplement: Supplemental data [file Supp_FileS2.docx]

**PubMed search strategy**

((Mindfulness-based coloring*[tw]) OR (Mandala painting*[tw]) OR (Mandala drawing*[tw]) OR (“Mandala-coloring” [tw]) OR (Mandala coloring*[tw]) OR (Deliberative coloring*[tw]) OR (coloring*[tw]) OR (Colouring*[tw]) OR (mandala art*[tw]) OR (mandala art therapy[tw]) OR (mandala*[tw]) OR ("Art therapy"[Mesh]) OR (Art therapy[tw]) OR (Structured coloring*[tw]) OR (Therapeutic coloring*[tw]) OR (Therapeutic artmaking*[tw]) OR (Art-based mandala*[tw]) OR (Painting*[tw]) OR (Drawing*[tw])) **AND** (("Stress, Psychological"[Mesh]) OR ("Stress, Physiological"[Mesh]) OR ("Depression"[Mesh]) OR ("Pain"[Mesh]) OR ("Fatigue"[Mesh]) OR ("Mindfulness"[Mesh]) OR ("Psychological Trauma"[Mesh]) OR ("Hope"[Mesh]) OR ("Resilience, Psychological"[Mesh]) OR (“mood”[tw]) OR (“well-being”[tw]) OR (“wellbeing”[tw]) OR (“flow”[tw]) OR ("Anxiety"[mesh]) OR ("Stress Disorders, Traumatic, Acute"[Mesh]) OR ("Stress Disorders, Traumatic"[Mesh]) OR ("Depressive disorder"[Mesh]) OR ("Psychological Distress"[Mesh]) OR(“Stress”[tw]) OR (“depression”[tw]) OR (“pain”[tw]) OR (“fatigue”[tw]) OR (“mindfulness”[tw]) OR (“trauma”[tw]) OR (“hope”[tw]) OR (“resilience”[tw]) OR (“anxiety”[tw]) OR (“distress”[tw])) **AND** ((patient*[tw]) OR ("Patients"[Mesh]) OR (cancer[tw]) OR (disability[tw]))
